# Profiles of 800,000 Users of the National Cancer Institute’s Cancer Information Service Since the Debut of Online Assistance, 2003-2008

**Published:** 2010-02-15

**Authors:** Nigel Bush, Robin Vanderpool, Ludmila Cofta-Woerpel, Phyllis Wallace

**Affiliations:** National Center for Telehealth and Technology, Defense Centers of Excellence for Psychological Health and Traumatic Brain Injury. At the time of this study, Dr Bush was affiliated with the Fred Hutchinson Cancer Research Center, Seattle, Washington; University of Kentucky, Lexington, Kentucky; University of Texas MD Anderson Cancer Center, Houston, Texas; Yale University, New Haven, Connecticut

## Abstract

**Introduction:**

In 2002, the Cancer Information Service (CIS) of the National Cancer Institute added to its toll-free telephone number 2 choices of media for access to cancer information specialists: e-mail and a proprietary online instant messaging service called LiveHelp. We sought to determine how new media users differ from telephone callers and the US population in general.

**Methods:**

During the 6 years since the new media were added, we collected data from more than 800,000 people who contacted CIS.

**Results:**

Telephone calls to CIS declined while the number of LiveHelp and e-mail inquiries steadily increased. People who contacted CIS by telephone and LiveHelp were predominantly white and female and, compared with the general population, were relatively well educated. LiveHelp users were significantly younger, more educated, and more affluent than telephone callers. CIS clients asked most frequently for general cancer site information, information about treatment and side effects management, screening programs, and economic assistance. Telephone callers most often asked about breast cancer.

**Conclusion:**

The Internet has introduced new sources of health information and possibly a new type of health information seeker. With LiveHelp and e-mail, CIS is poised to meet the needs of the digital health consumer and also the traditional telephone caller.

## Introduction

Cancer Information Service (CIS), a program of the National Cancer Institute (NCI) (1), is NCI's link to the public; it presents cancer-related information and research findings in a clear and understandable manner. Since its inception in 1975, CIS has handled more than 10 million inquiries from patients and their families, members of the public, and health professionals regarding cancer prevention, risk factors, early detection, symptoms, diagnosis, treatment, and research.

The CIS fulfills its mission through a 3-pronged approach: a community-based partnership program (2), a collaborative research program (3), and the information service (4). For most of its history, CIS's national toll-free 1-800-4-CANCER telephone line has been the primary conduit for information seekers. Callers receive assistance with their inquiries from highly trained information specialists at CIS contact centers.

By 2000, however, searching for health information was among the most popular uses of the Internet (5,6). In response, CIS analyzed more than 6,000 telephone callers to its service to assess their Internet use and interest in such technologies to access health information (7). Results showed that by 2002, 40% of CIS callers also used the Internet to obtain cancer information, but only half of those Internet users found all the information they needed online; one-third called CIS specifically to discuss or have explained the information they found on the Internet. The CIS surveys showed a lack of authoritative, comprehensive, up-to-date, and easily understandable cancer information online. Consequently, in 2002, CIS introduced 2 online channels for access to CIS cancer information specialists: LiveHelp (8) and e-mail. LiveHelp is the Web-based, instant messaging equivalent of the telephone service. It allows a real-time online dialogue, including linking to resources, between the user and an information specialist. Information specialists also field inquiries via e-mail.

Since the inauguration of our online media, CIS has fielded more than 800,000 inquiries by telephone, LiveHelp, and e-mail. For our service to remain vital and relevant, we need to understand and respond to the changing cancer information and communication needs of our clientele. Our research questions, referring to the period from January 2003 through December 2008, were as follows: 1) What was the volume of inquiries to CIS from each of the 3 communications media and how did those relative contact volumes change over time? 2) What were the sociodemographic characteristics of our clients and how did they change over time? 3) How did the demographic characteristics of CIS clientele compare with those of the general population and with other national estimates of health information seekers? 4) Were there regional or geographic variations in call or contact volumes? 5) How did our traditional telephone callers compare with users of the new online media? The answers to each of those questions will enable us to identify and provide help to the widest possible population.

## Methods

### Data collection

CIS collects detailed standardized data from clients with approval from the contact center institutional review board and the federal Office of Management and Budget. Since 1998, information specialists have used a proprietary Web-accessible electronic contact record form (ECRF) to document their interactions with clients and to capture client information. Similar ECRFs are used for telephone and LiveHelp inquiries. During the open-ended conversation with the client (verbal for telephone and typed online in real-time for LiveHelp), information specialists complete pertinent text and notes fields, select responses from contextually generated drop-down lists, search extensive NCI databases, and connect to online materials ordering services from the ECRF screens. Any remaining data fields are completed immediately after the end of the call or instant messaging.

Many data are collected passively during the interaction with the client. Information, including type of seeker or client, subject of interaction, type of cancer, CIS response to the caller's inquiry, and resources used are collected from all clients. Information specialists also actively ask all clients how they found out about the CIS, if they had contacted the CIS before, and for their zip code. Additional demographic information, including age, sex, education level, ethnicity and race, household income, sources of health care, and insurance status, is collected on a random sample varying from a routine 25% of clients to 100% periodically for special national or regional promotions and research projects.

Procedures for addressing e-mail inquiries differ from LiveHelp and telephone inquiries. A draft response is prepared for each e-mail, which is reviewed internally, and a final response is compiled by using a CIS e-mail template. Once the e-mail response is sent to the client, an ECRF is completed after the fact on the basis of the text of the CIS response. Because of Office of Management and Budget restrictions, demographic data, including sex, race, and education, are not collected from CIS e-mail users.

Accuracy and consistency of ECRFs among information specialists are maintained by a rigorous quality assurance process. Daily samples of audio files, LiveHelp transcripts, and e-mail content are compared with respective ECRF records by internal and national CIS project office supervisors, and regular feedback is provided.

### Comparative data

We compared various ECRF demographic data with their equivalents from available national surveys, specifically with US census data for the study period (9) and with more focused information about the demographics of cancer information seekers from 3 successive Health Information National Trend Surveys (HINTS) in 2003, 2005, and 2007. HINTS routinely collects nationally representative data about the American public's use of cancer-related information (10). HINTS collects data by random-digit-dial telephone surveying in all 50 states by using a computer-assisted telephone interview format to accommodate complex skip-patterns. Interviewers follow an on-screen sampling algorithm to select a single sampled person from all adults aged 18 years or older in the household. Survey administration averages 30 minutes per respondent. The most recent survey additionally collected equivalent data by mailed printed questionnaire. In the general HINTS framework, specific questions have differed in the 3 surveys to date. In our comparisons, we matched the most recent available HINTS survey questions with their corresponding ECRF and census items from the same period, ideally from 2007 but in some instances from 2005 or 2003.

### Data analysis

ECRF data collected at CIS contact centers in New York, Miami, and Seattle are contained in a centralized database at NCI. Data presented here were cleaned and consolidated into an SPSS version 16 (SPSS Inc, Chicago, Illinois) data set for analysis. For calculations of significance, we first tested selected distributions for normality with the Kolmogorov-Smirnoff procedure and then applied the appropriate parametric or nonparametric test as indicated.

## Results

**Figure 1:**
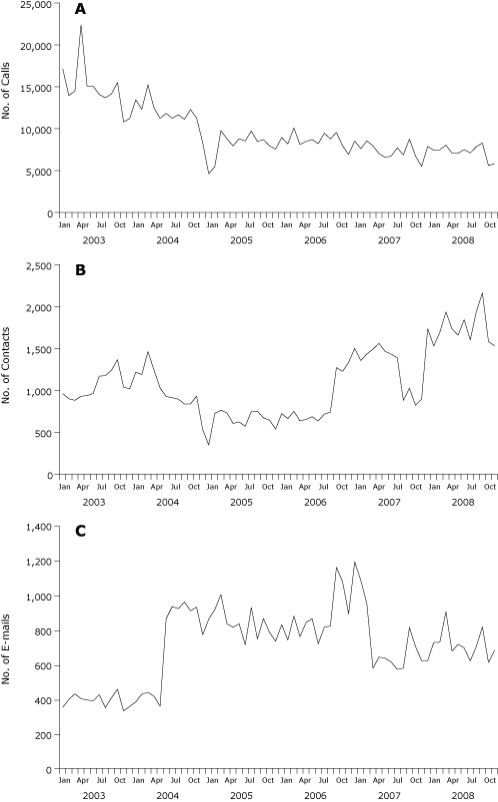
Number of calls (A), LiveHelp contacts (B), and e-mails (C) to the National Cancer Institute's Cancer Information Service, 2003-2008.

The final 2003-2008 ECRF data set used here represents 825,869 inquiries to CIS. From January 2003 through December 2008, combined inquiries from all media to CIS declined from approximately 18,500 to 8,000. These figures were dominated by telephone inquiries; the number of queries to LiveHelp and e-mail were an order of magnitude smaller throughout (Figure 1), although telephone calls steadily declined while LiveHelp and e-mail use nearly doubled.

**Table:** 

**Count**			
**Months 2003-2008**	**Telephone**	**E-mail**	**LiveHelp**
Jan	17130	354	961
Feb	13966	399	899
Mar	14512	432	881
Apr	22407	404	929
May	15077	396	937
Jun	15085	392	963
Jul	14098	427	1169
Aug	13701	352	1175
Sep	14157	409	1239
Oct	15517	458	1366
Nov	10808	333	1038
Dec	11230	359	1018
Jan	13457	385	1216
Feb	12320	430	1189
Mar	15250	440	1462
Apr	12482	418	1242
May	11242	360	1028
Jun	11839	866	927
Jul	11257	936	912
Aug	11674	923	895
Sep	11131	962	837
Oct	12297	911	837
Nov	11301	932	932
Dec	8349	773	534
Jan	4630	862	346
Feb	5486	918	724
Mar	9791	1005	763
Apr	8800	836	730
May	7957	816	605
Jun	8800	836	623
Jul	8541	715	571
Aug	9744	930	747
Sep	8497	749	750
Oct	8710	866	672
Nov	7956	786	643
Dec	7569	736	537
Jan	8969	831	723
Feb	8195	743	662
Mar	10098	881	750
Apr	8128	762	636
May	8487	843	652
Jun	8710	866	685
Jul	8222	720	634
Aug	9470	816	716
Sep	8788	823	740
Oct	9563	1162	1272
Nov	7979	1080	1226
Dec	6937	893	1333
Jan	8539	1194	1501
Feb	7621	1087	1355
Mar	8568	952	1434
Apr	7954	579	1490
May	7047	644	1561
Jun	6573	638	1465
Jul	6752	616	1431
Aug	7711	573	1389
Sep	6895	580	879
Oct	8767	815	1025
Nov	6733	705	823
Dec	5521	623	894
Jan	7899	624	1737
Feb	7432	728	1530
Mar	7496	730	1693
Apr	8047	906	1935
May	7094	678	1734
Jun	7079	718	1658
Jul	7518	697	1843
Aug	7121	622	1602
Sep	7870	705	1939
Oct	8301	818	2163
Nov	5602	613	1581
Dec	5842	683	1531

We examined the possibility of regional variations in CIS inquiries by calculating the incidence of contacts to CIS per 100,000 of each state's population for each month and year of the study. Although these analyses revealed regional spikes in incidence of contacts from month to month, these were inconsistent and transient with 2 exceptions. Washington, DC, and Maryland consistently had more contacts per capita to CIS than any other state or region.

By most ECRF measures, callers to CIS at the end of our study period were similar to callers at the beginning (Table 1). In 2003 and 2008, callers were predominantly female, overwhelmingly white (the largest minority was African American), well educated (more than one-third were college graduates), most commonly calling about breast cancer, and typically inquiring about general cancer site information, treatment and side effects management, screening programs, and economic assistance.

LiveHelp users had much in common with telephone callers. As with telephone callers, LiveHelp users were mostly white women who asked most frequently for general cancer site information, treatment and side effects management, screening programs, and economic assistance, and in particular, about breast cancer (Table 1). Our more limited e-mail data show that inquiries from e-mail users generally conformed to those by telephone and LiveHelp. Perhaps the most notable exception was the higher proportion of e-mail users who were health professionals compared with telephone and LiveHelp users.

Although HINTS found that the percentage of female health information seekers was high, our percentage of female CIS telephone and LiveHelp users was higher still and much higher than the percentage of women in the general population and in other studies (Table 2) (11).

Proportions of white and American Indian/Alaska Native CIS clients generally resembled those of HINTS respondents and the general US population; 80% of telephone callers and 85% of LiveHelp users during the 6 study years were white. Relative to HINTS respondents and the general US population, African Americans were overrepresented among telephone callers (13%) and underrepresented among LiveHelp users (6%), and Asians were overrepresented among LiveHelp users (7%).

CIS clients tended to be more educated than the population in general, offline health information seekers (11), and Internet users in general (12). CIS telephone callers were more represented in the lowest income category and less in the highest income category compared with LiveHelp users, HINTS respondents, or the general population.

**Figure 2:**
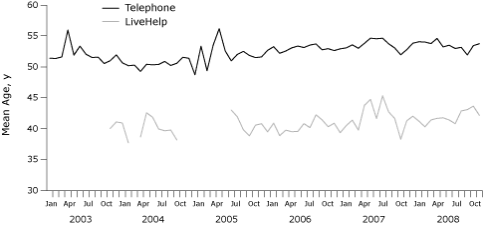
Mean age of telephone callers and LiveHelp users of the National Cancer Institute's Cancer Information Service, 2003-2008. Telephone callers (mean age, 52 y; standard deviation, 15.4 y) were significantly older than LiveHelp users (mean age, 41 y; standard deviation, 13.2 y) across all 6 years (independent samples *t* test, *P* < .001). For some periods during reorganization of Cancer Information Service infrastructure (breaks in the red line above), age was not collected in LiveHelp.

**Figure 3:**
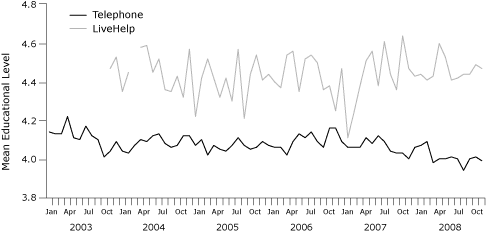
Mean educational level of telephone callers and LiveHelp users of the National Cancer Institute's Cancer Information Service, 2003-2008. We classified educational level on a 6-point scale as follows: 1, grade school; 2, some high school; 3, high school graduate; 4, some college; 5, college graduate; and 6, postgraduate. LiveHelp users reported significantly more education across all 6 years (independent samples Mann-Whitney U test, *P* < .001) than did telephone callers. For some periods during reorganization of Cancer Information Service infrastructure (breaks in the red line above), educational level was not collected in LiveHelp.

**Figure 4:**
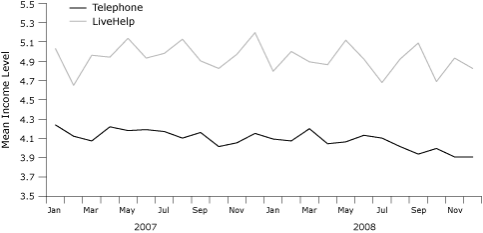
Mean household income of telephone callers and LiveHelp users of the National Cancer Institute's Cancer Information Service, 2007 and 2008. We classified household income on a 7-point scale as follows: 1, <$10,000; 2, $10,000-$19,000; 3, $20,000-$29,000; 4, $30,000-$39,000; 5, $40,000-$59,000; 6, $60,000-$79,000; and 7, ≥$80,000. LiveHelp users reported significantly more income than did telephone callers both years (independent samples Mann-Whitney U test, *P* < .001). Income data were not collected before January 2007

LiveHelp users were significantly younger than telephone callers (Figure 2). LiveHelp users were also significantly more educated than telephone callers (Figure 3). Forty-nine percent of LiveHelp users were college graduates, compared with 36% of telephone callers. LiveHelp users also reported significantly higher income than did telephone callers (Figure 4). For example, 46% of LiveHelp users reported annual household incomes of more than $60,000, compared with only 29% of telephone callers.

**Table:** 

**Mean age by month by access**		
**Months 2003-2008**	**Telephone**	**LiveHelp**
Jan	51.42	
Feb	51.38	
Mar	51.63	
Apr	55.98	
May	51.92	
Jun	53.38	
Jul	52.06	
Aug	51.54	
Sep	51.59	
Oct	50.57	
Nov	51.02	40.06
Dec	51.97	41.12
Jan	50.68	40.95
Feb	50.21	37.73
Mar	50.31	
Apr	49.29	38.68
May	50.44	42.60
Jun	50.36	41.88
Jul	50.42	39.98
Aug	50.90	39.69
Sep	50.25	39.81
Oct	50.61	38.21
Nov	51.57	
Dec	51.41	
Jan	48.75	
Feb	53.36	
Mar	49.40	
Apr	53.50	
May	56.20	
Jun	52.60	
Jul	50.99	43.04
Aug	52.04	41.95
Sep	52.53	39.84
Oct	51.87	38.87
Nov	51.52	40.60
Dec	51.63	40.83
Jan	52.70	39.52
Feb	53.28	40.92
Mar	52.22	38.90
Apr	52.59	39.79
May	53.10	39.54
Jun	53.39	39.58
Jul	53.15	40.85
Aug	53.55	40.20
Sep	53.73	42.27
Oct	52.78	41.46
Nov	52.97	40.35
Dec	52.66	40.91
Jan	52.94	39.38
Feb	53.10	40.53
Mar	53.59	41.41
Apr	53.03	39.81
May	53.83	43.81
Jun	54.66	44.76
Jul	54.57	41.67
Aug	54.66	45.34
Sep	53.76	42.74
Oct	53.08	41.69
Nov	51.98	38.33
Dec	52.81	41.31
Jan	53.85	42.04
Feb	54.08	41.25
Mar	54.04	40.33
Apr	53.79	41.44
May	54.62	41.70
Jun	53.25	41.79
Jul	53.52	41.44
Aug	53.00	40.83
Sep	53.18	42.90
Oct	51.95	43.11
Nov	53.46	43.67
Dec	53.77	42.17

**Table:** 

**Mean educational level by month by access**		
**Months 2003-2008**	**Telephone**	**LiveHelp**
Jan	4.12	
Feb	4.11	
Mar	4.11	
Apr	4.20	
May	4.09	
Jun	4.08	
Jul	4.15	
Aug	4.10	
Sep	4.08	
Oct	3.99	
Nov	4.02	4.45
Dec	4.07	4.51
Jan	4.02	4.33
Feb	4.01	4.43
Mar	4.05	
Apr	4.08	4.56
May	4.07	4.57
Jun	4.10	4.43
Jul	4.11	4.50
Aug	4.06	4.34
Sep	4.04	4.33
Oct	4.05	4.41
Nov	4.10	4.30
Dec	4.10	4.55
Jan	4.05	4.20
Feb	4.08	4.40
Mar	4.00	4.50
Apr	4.05	4.40
May	4.03	4.30
Jun	4.02	4.40
Jul	4.05	4.28
Aug	4.09	4.55
Sep	4.05	4.19
Oct	4.03	4.42
Nov	4.04	4.52
Dec	4.07	4.39
Jan	4.05	4.42
Feb	4.04	4.38
Mar	4.04	4.35
Apr	4.00	4.52
May	4.07	4.54
Jun	4.11	4.33
Jul	4.09	4.50
Aug	4.12	4.52
Sep	4.07	4.48
Oct	4.04	4.34
Nov	4.14	4.36
Dec	4.14	4.23
Jan	4.07	4.45
Feb	4.04	4.09
Mar	4.04	4.22
Apr	4.04	4.36
May	4.09	4.49
Jun	4.06	4.54
Jul	4.10	4.36
Aug	4.07	4.59
Sep	4.02	4.42
Oct	4.01	4.34
Nov	4.01	4.62
Dec	3.98	4.45
Jan	4.04	4.41
Feb	4.05	4.42
Mar	4.07	4.39
Apr	3.96	4.41
May	3.98	4.58
Jun	3.98	4.51
Jul	3.99	4.39
Aug	3.98	4.40
Sep	3.92	4.42
Oct	3.98	4.42
Nov	3.99	4.47
Dec	3.97	4.45

**Table:** 

Mean income level by month by access		
	**Telephone**	**LiveHelp**
Jan	4.21	5.02
Feb	4.09	4.63
Mar	4.04	4.95
Apr	4.19	4.93
May	4.15	5.13
Jun	4.16	4.92
Jul	4.14	4.97
Aug	4.07	5.12
Sep	4.13	4.89
Oct	3.98	4.81
Nov	4.02	4.96
Dec	4.12	5.19
Jan	4.06	4.78
Feb	4.04	4.99
Mar	4.17	4.88
Apr	4.01	4.85
May	4.03	5.11
Jun	4.10	4.91
Jul	4.07	4.66
Aug	3.98	4.91
Sep	3.90	5.08
Oct	3.96	4.67
Nov	3.87	4.92
Dec	3.87	4.81

## Discussion

### Decrease in telephone calls and increase in LiveHelp and e-mail inquiries

The number of telephone calls to CIS decreased significantly from 2003 through 2008, but the increase in new media users was too small to account for the entire decline. This decrease in telephone calls, however, corresponded to the increase in US adults who chose the Internet in general as their preferred source of health information (13). A series of Harris polls in the last 10 years showed a dramatic increase in US adults who were online (from 38% in 1998 to nearly 80% by 2008) and an equally dramatic increase in adults who looked online for health information (from 27% to 66% during the same period) (14). HINTS found that 58% of respondents looked for health or medical information on the Internet for themselves, and 60% had searched for others (10). HINTS also reported that nearly 28% of respondents used the Internet as their preferred source of cancer information, and only 6% chose to contact an information specialist. Pew Internet surveys from 2002 through 2008 consistently showed that 75% to 80% of Internet users looked online for health information (12).

The increasing availability of health information online coincided with the decline in telephone calls to CIS, which suggests a causal connection. The increase in use of CIS's online LiveHelp and e-mail services also followed the same pattern; however, improvements in CIS workforce management and technology may have contributed to increased efficiency of the new services.

### Awareness of CIS

As a public service, CIS has never designated funds for self-promotion. Consequently, a lack of awareness of CIS among patients, the public, and health professionals has frustrated the service since its inception. HINTS surveys found that 75% of respondents had heard of NCI, but only 28% had heard of CIS, and only 19% had heard of the CIS 800 telephone number (10,15). When we mapped regional incidence of contacts to CIS for individual years, we found periodic spikes in per capita volume for various states that we could attribute to regional cancer awareness campaigns, media stories, or other events that temporarily stimulated a local swell in cancer inquiries.

We also found a higher rate of contacts from the Washington, DC/Maryland area across all months, independent of awareness campaigns or other events, which suggests that public awareness of CIS is high in that region. Residents of that region may be more aware of the National Institutes of Health in Bethesda, Maryland, which includes the NCI's CIS. Residents may be more likely to turn to the familiar federal resource than are information seekers beyond the metropolitan area.

### Demographic characteristics of CIS clients

Since 2003, the demographic profile of the typical CIS client has not changed. Younger age, higher educational attainment, and higher socioeconomic status are positively related to active information seeking (16-18), which is consistent with the makeup of typical (younger, more educated, and more affluent) LiveHelp users. CIS telephone users are also more educated than are comparable populations from national surveys, which may indicate that, unfortunately, only well-informed people access CIS.

LiveHelp users were significantly younger, more educated, and more affluent than were CIS callers. Income is the primary factor in the disparity in use of online services (19-27); in fact, income appears to predict Internet access even more than race and ethnicity do (28). LiveHelp users reported household incomes generally representative of the US population, but CIS callers were less affluent. The literature consistently shows barriers to Internet use for people with less education (13,26,29,30), which is consistent with the fact that substantially more LiveHelp users were college graduates than in the general population.

Several factors may explain the effect of new media on the use of CIS services. Some CIS telephone clients may have switched to LiveHelp or e-mail once they became available, which would partially account for the decline in telephone calls. Another explanation could be that new media attracted a young, affluent, educated clientele who otherwise would not have called CIS, and the numbers of telephone callers may have declined for other reasons. Most likely, a combination of these factors resulted in the decline in telephone inquiries, which indicates a social trend in information seeking — a general turn away from traditional services such as the CIS 800 line and toward the Internet as the first choice for health information.

### Limitations

These data are primarily descriptive and derived from client self-report. Although our quality assurance procedures ensure high reliability of data gathering, those data are subject to the inconsistencies of self-report. Our analysis and discussion lack a theoretical component, but CIS was not driven by theoretical models at its inception. Initially, CIS was established in response to the National Cancer Act, and points of access were added on the basis of feedback from the public.

### Conclusions

The fact that most people are not aware of CIS is its biggest challenge. Public organizations such as CIS need to be able to apply funds toward promotional efforts, much in the way the American Cancer Society has successfully done, but this cannot happen without a federal reexamination of policy. Although we have seen a fundamental shift in the last 6 years in the way people seek health information, the telephone remains the medium of choice for contacting CIS. The CIS telephone service provides a haven for older adults and racial and ethnic minorities who are seeking information but may not have access to the Internet.

Some information seekers may prefer the Internet for its anonymity, but many more need the reassurance of interacting with a person. CIS's telephone service has always offered individualized, tailored, flexible relationships with clients who commonly are coping with life-altering medical conditions. First-hand reports indicate that cancer patients and family members value personal rapport with the information specialist and appreciate the accuracy and legitimacy of the information CIS provides (31). In time, LiveHelp instant messaging may become the standard for a real-time online supplement, if not alternative, to health information given over the telephone.

## Figures and Tables

**Table 1:** Selected Characteristics of Users of the National Cancer Institute's Cancer Information Service, by Method of Contact, 2003 and 2008

Characteristic	% of Contacts					

	Telephone		LiveHelp		E-mail	

	2003	2008	2003	2008	2003	2008
**Sex**						
Female	74	76	76	76	NC	
**Race/ethnicity**						
White	83	77	84	84	NC	
African American	12	17	7	5		
Hispanic	10	12	5	8		
Asian	2	2	8	8		
**Education**						
College graduate or higher	38	35	50	51	NC	
**Category of inquiry**						
General cancer site information	20	18	30	32	20	21
Treatment/side effects management	11	12	17	15	12	20
Hospital/clinic screening programs	11	17	4	7	4	5
Economic assistance	7	11	3	4	4	6
**Cancer topic of inquiry**						
Breast	26	27	20	20	22	21
Colorectal	7	8	7	6	6	6
Prostate	8	8	6	7	7	7
Lung	19	9	9	9	9	7
**Type of client**						
General public	41	26	30	27	26	29
Patient in treatment	9	12	6	5	3	5
Health professional	5	5	6	4	16	9
Family/friend of patient	29	35	41	41	32	38

Abbreviation: NC, not collected.

**Table 2:** Selected Characteristics of Users of the National Cancer Institute's Cancer Information Service (CIS), Respondents to the Health Information National Trend Surveys (HINTS), and the General US Population

**Characteristic**	% of CIS Telephone Callers	% of CIS LiveHelp Users	% of HINTS (10) Cancer Information Seekers	% of US Population (9)
**Sex**				
Female	76	75	60	51
**Ethnicity**				
Hispanic	11	9	9	12
**Race**				
White	79	85	76	75
African American	15	5	8	12
American Indian/Alaska Native	4	2	2	1
Asian	3	8	2	4
**Education**				
College graduate or higher	40	49	31	24
**Total household income^a^**				
<$39,000^b^ or <$35,000^c^	55^b^	35^b^	31^c^	33^c^
$39,000-$80,000^b^ or $35,000-$75,000^c^	26^b^	32^b^	30^c^	30^c^
>$80,000^b^ or >$75,000^c^	19^b^	32^b^	29^c^	28^c^

Abbreviation: HINTS, Health Information National Trends Surveys.

a Exact category matches for CIS income and HINTS/US population income were not available. The categories shown are the closest corresponding income ranges collected by CIS,

b HINTS,

c and the US Census.
